# Developing mRNA signatures as a novel prognostic biomarker predicting high risk multiple myeloma

**DOI:** 10.3389/fonc.2023.1105196

**Published:** 2023-02-23

**Authors:** Jing Wang, Lili Guo, Chenglan Lv, Min Zhou, Yuan Wan

**Affiliations:** ^1^ Department of Oncology and Hematology, Yizheng Hospital of Nanjing Drum Tower Hospital Group, Yizheng, China; ^2^ Department of Hematology, The Affiliated Drum Tower Hospital of Nanjing University Medical School, Nanjing, China; ^3^ The Pq Laboratory of BiomeDx/Rx, Department of Biomedical Engineering, Binghamton University, State University of New York (SUNY), Binghamton, NY, United States; ^4^ The Second Hospital of Nanjing, Nanjing University of Chinese Medicine, Nanjing, China

**Keywords:** myeloma, data processing, drug response prediction, prognostic model, RNA-seq

## Abstract

**Background:**

Multiple myeloma (MM) remains an essentially incurable disease. This study aimed to establish a predictive model for estimating prognosis in newly diagnosed MM based on gene expression profiles.

**Methods:**

RNA-seq data were downloaded from the Multiple Myeloma Research Foundation (MMRF) CoMMpass Study and the Genotype-Tissue Expression (GTEx) databases. Weighted gene coexpression network analysis (WGCNA) and protein-protein interaction network analysis were performed to identify hub genes. Enrichment analysis was also conducted. Patients were randomly split into training (70%) and validation (30%) datasets to build a prognostic scoring model based on the least absolute shrinkage and selection operator (LASSO). CIBERSORT was applied to estimate the proportion of 22 immune cells in the microenvironment. Drug sensitivity was analyzed using the OncoPredict algorithm.

**Results:**

A total of 860 newly diagnosed MM samples and 444 normal counterparts were screened as the datasets. WGCNA was applied to analyze the RNA-seq data of 1589 intersecting genes between differentially expressed genes and prognostic genes. The blue module in the PPI networks was analyzed with Cytoscape, and 10 hub genes were identified using the MCODE plug-in. A three-gene (TTK, GINS1, and NCAPG) prognostic model was constructed. This risk model showed remarkable prognostic value. CIBERSORT assessment revealed the risk model to be correlated with activated memory CD4 T cells, M0 macrophages, M1 macrophages, eosinophils, activated dendritic cells, and activated mast cells. Furthermore, based on OncoPredict, high-risk MM patients were sensitive to eight drugs.

**Conclusions:**

We identified and constructed a three-gene-based prognostic model, which may provide new and in-depth insights into the treatment of MM patients.

## Introduction

Multiple myeloma (MM) is the second most common malignant hematological disease, accounting for approximately 10% of all hematologic malignancies (1). Our understanding of the biological mechanisms underlying the development of MM has advanced greatly. Survival in multiple myeloma has improved significantly in the last decade (2), but MM remains an essentially incurable disease. It is therefore of great clinical importance to find novel molecular markers for molecular targeted treatment of MM. MM is an invariably fatal disease with a highly heterogeneous outcome because of heterogeneous genomes. Transcriptome and proteome maps will accelerate the discovery of new therapeutic targets based on disease biology and the identification of biomarkers to guide therapeutic decisions in MM. Gene expression profiling (GEP) is a useful tool to estimate the aggressiveness of MM and will help to make individualized therapeutic decisions (3). Many different gene expression-based prognostic signatures have been reported for MM in the last decade (4–8). In 2011, the Multiple Myeloma Research Foundation (MMRF) CoMMpass Study was initiated, which gathered information on close to 1200 patients aged 27 to 93 years and followed up on a biannual basis for at least 8 years (9). The main objectives of our study were to establish universal prognostic gene signatures to enable the stratification of newly diagnosed MM patients at higher risk based on the MMRF CoMMpass study, which may provide new biomarkers serving as druggable targets for the treatment of high-risk MM patients.

## Materials and methods

### Data acquisition and preprocessing

We obtained the RNA-seq transcriptome data of 860 newly diagnosed MM samples and 444 normal samples from the MMRF CoMMpass and Genotype-Tissue Expression (GTEx) database. Normalized read counts to these assemblies were calculated using transcripts per kilobase million (TPM) values. Clinical information (age, sex, ISS stage, survival time and status) was also collected from MMRF. Then, the differentially expressed genes (DEGs) were selected by using the “limma” package (10) with | log FC | ≥ 1 and p value < 0.05.

### Identification of prognostic genes

Through univariate Cox analysis, the association between expression levels of genes and MM patients’ overall survival (OS) was explored. Genes with P < 0.05 based on the univariate analysis were identified as PGs. A Venn diagram was drawn to select intersecting genes between PGs and DEGs.

### Functional enrichment analysis

Gene Ontology (GO) functional enrichment analysis was performed using the R package “clusterProfiler” (11).

### Weighted gene co-expression network analysis

The construction of the gene coexpression network was completed using the “WGCNA” package (12) to explore the correlation of intersecting genes and search for important interacting gene modules. The correlation of gene expression profiles and module eigengenes was represented by module membership.

### Generation and analysis of a protein-protein interaction network

A PPI network of module eigengenes was constructed using the String database (https://www.string-db.org/). Molecular Complex Detection (MCODE) in Cytoscape (Version 3.8.0,RRID : SCR_003032) was applied to screen hub genes with MCODE score >3 and number of nodes >4.

### Construction and verification of the risk model

A total of 756 patients with MM were randomly split into a training cohort (n = 530) and a validation cohort (n = 226). LASSO Cox performs collinearity processing on the filtered Hub genes. The coefficient of more important survival-associated genes is determined when the adding appropriate penalty (lambda) is the minimum. An optimal cutoff was identified *via* the method of maximally selected rank statistics to develop a prognosis classifier for MM patients in training cohort. The GEO database, including GSE4581 and GSE57317, validated the prognostic model. A receiver operating characteristic (ROC) curve was applied to evaluate the predictive performance of our risk model compared with other models (4–6).

### Construction and prediction of the nomogram

A clinical nomogram was developed to predict OS using the “rms” package. The calibration curve was applied to evaluate the consistency between the nomograms. Decision curve analysis (DCA) was used to evaluate the nomogram.

### Gene set enrichment analysis (RRID : SCR_003199)

Reactome GSEA pathway (13) interaction analysis was run to compare the gene expression profiles of different risk groups.

### Tumor-infiltrating immune cell analysis

The relative abundance of 22 TIIC subpopulations was estimated by applying CIBERSORT (14) in the MM high- and low-risk groups.

### Predictions for drug sensitivity analysis

The R package “OncoPredict” (15) of 198 drugs was used to predict *in vivo* drug responses in high-risk MM patients.

### Statistical analysis

All statistical analyses were performed using packages developed in R 4.1.1 (R Project for Statistical Computing, RRID : SCR_001905). A p value of less than 0.05 was used for statistical significance.

## Results

### Identification of DEGs

**Figure 1:**
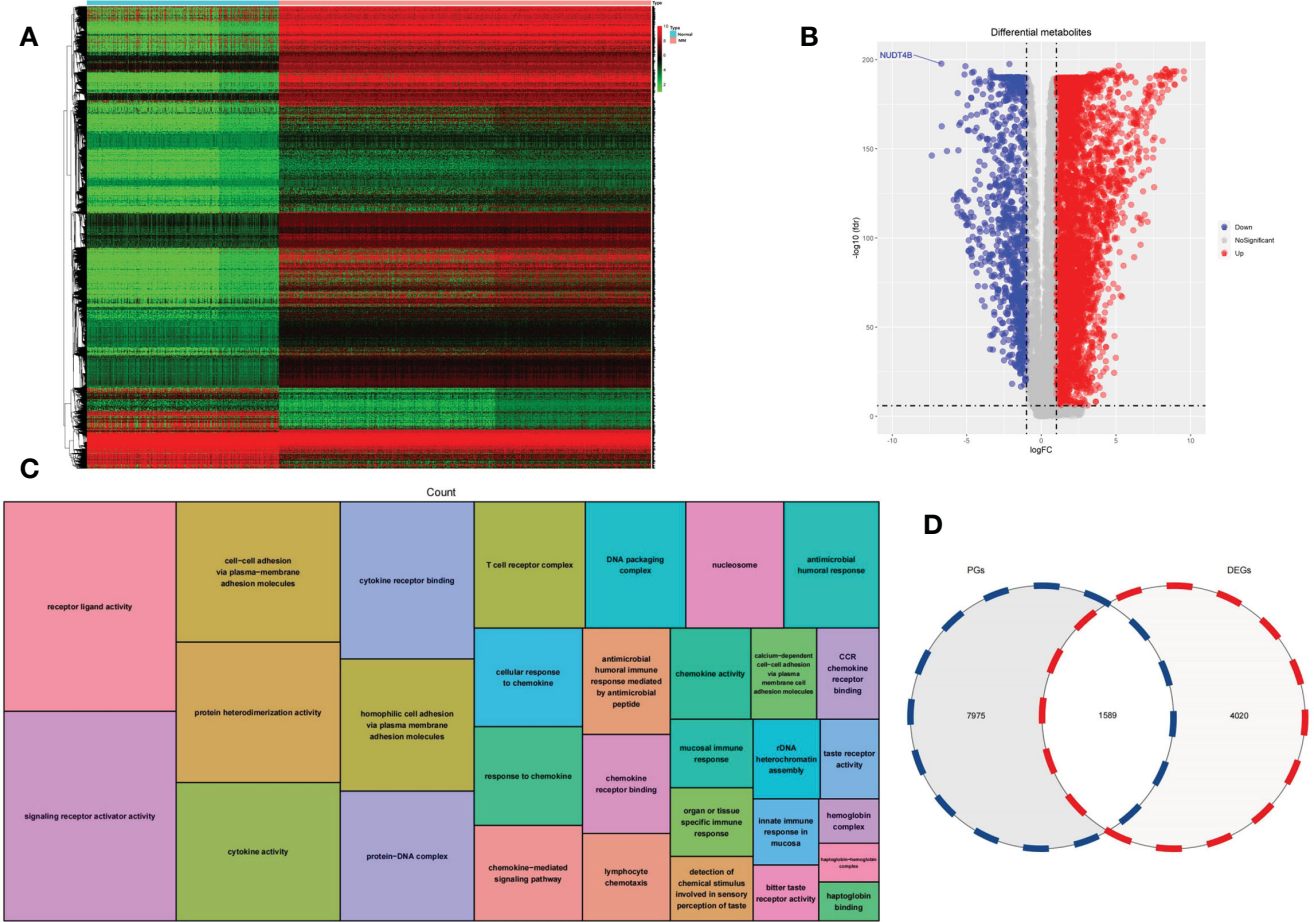
**(A)** Heatmap was used to visualize DEGs. **(B)** A volcano plot was generated to visualize DEGs. **(C)** GO functional enrichment analysis of DEGs. **(D)** A Venn diagram was used to identify intersecting genes between PGs and DEGs.

A heatmap of DEGs according to gene expression in 860 MM samples and 444 normal counterparts is shown in **Figure 1A**. In total, 4672 overexpressed genes and 937 underexpressed genes were identified (**Figure 1B**). GO functional enrichment analysis of DEGs is shown in **Figure 1C**.

### Intersecting genes between DEGs and PGs

We analyzed the correlation between the expression of each gene and the overall survival of MM patients to find 9564 PGs. A total of 1589 genes were classified as intersecting genes between DEGs and PGs (**Figure 1D**).

### WGCNA

**Figure 2:**
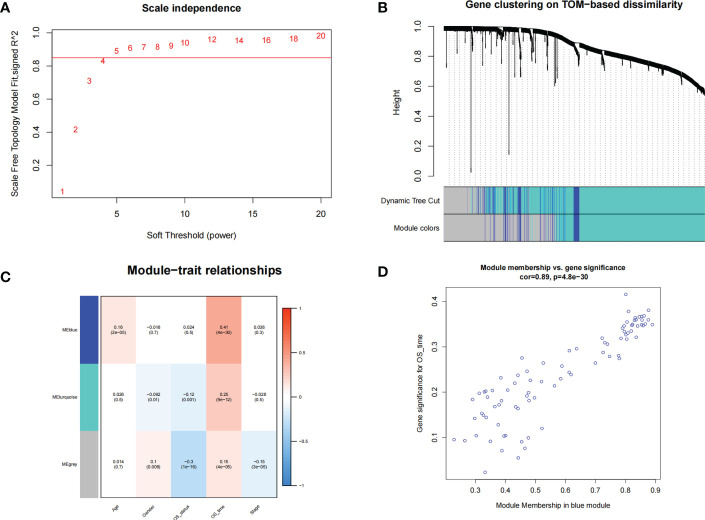
WGCNA. **(A)** The soft threshold power of WGCNA was determined based on the scale-free fitting index R2. **(B)** Clustering dendrograms of genes based on coexpression network analysis. **(C)** A heatmap showing the correlation between gene modules and clinical features. **(D)** A scatter plot of gene significance versus the module membership in the blue module.

A total of 1589 intersecting genes were included in WGCNA. The soft threshold of β=5 was identified to construct a scale-free network (**Figure 2A**). Under the clustering criteria of mergecutheight and minModuleSize of 0.25 and 30, respectively, a total of 2 modules (blue and turquoise) were obtained (**Figure 2B**). Module blue was the most relevant module for trait (**Figures 2C, D**).

### PPI network construction and hub gene selection

**Figure 3:**
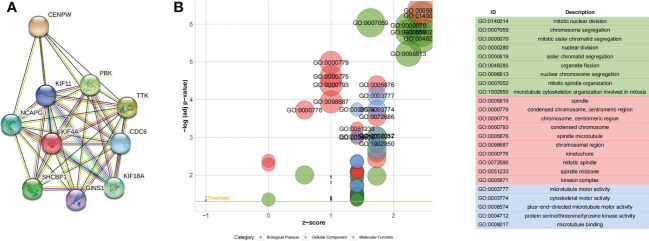
**(A)** Ten hub genes identified by the MCODE plug-in. **(B)** GO functional enrichment analysis of hub genes.

A total of 119 genes found in the blue module were imported into the STRING database to obtain the interaction relationships. In total, there were 10 hub genes from the blue modules using the MCODE plug-in of Cytoscape for subsequent analysis. Visualization results are shown in **Figure 3A**. GO analysis showed that the hub genes were mainly involved in mitotic nuclear division, as illustrated in **Figure 3B**.

### Risk prediction model construction and validation

**Figure 4:**
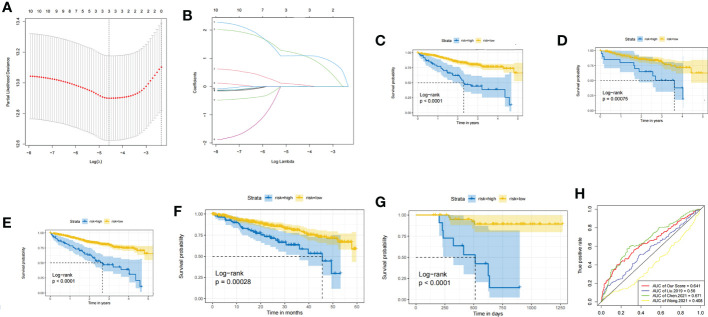
**(A)** LASSO for risk factor screening. **(B)** LASSO variable trajectory diagram. **(C)** KM curves of the training set. **(D)** KM curves of the validation set. **(E)** KM curves of the whole MMRF CoMMpass. **(F)** KM curves of the GSE4581 cohort. **(G)** KM curves of the GSE57317 cohort. **(H)** ROC curve for validation.

In the training set, the dimensionality reduction of hub genes was implemented by Lasso regression, as presented in **Figures 4A, B**, and a prognostic model was constructed based on three genes (TTK, GINS1, and NCAPG). The identified risk scoring equation is as follows: Risk score = TTK×0.0770053593671592+ GINS1×1.1810301963703+ NCAPG×1.09399274693476. There were significant differences between the survival of patients with different risks in the training set, validation set and whole MMRF cohort (p < 0.001, **Figures 4C-E**). The same results are shown in GSE4581 cohort and GSE57317 cohort (**Figures 4F, G**). ROC curves were applied for the prediction accuracy of different models (**Figure 4H**).

### Nomogram construction and validation

**Figure 5:**
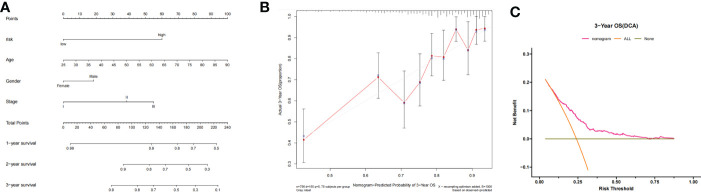
**(A)** Nomogram-based prognosis prediction model. **(B)** Calibration curve of the nomogram. **(C)** Decision curve analysis of the nomogram.

A nomogram was constructed according to the contributions of age, sex, ISS staging, and risk status, as shown in **Figure 5A**. The risk status was evaluated by the risk scoring equation. The calibration curve showed that the predicted probability of the nomogram was consistent with the actual observed probability (**Figure 5B**). The DCA curve demonstrated that the nomogram was clinically useful (**Figure 5C**).

### TIIC analysis

**Figure 6:**
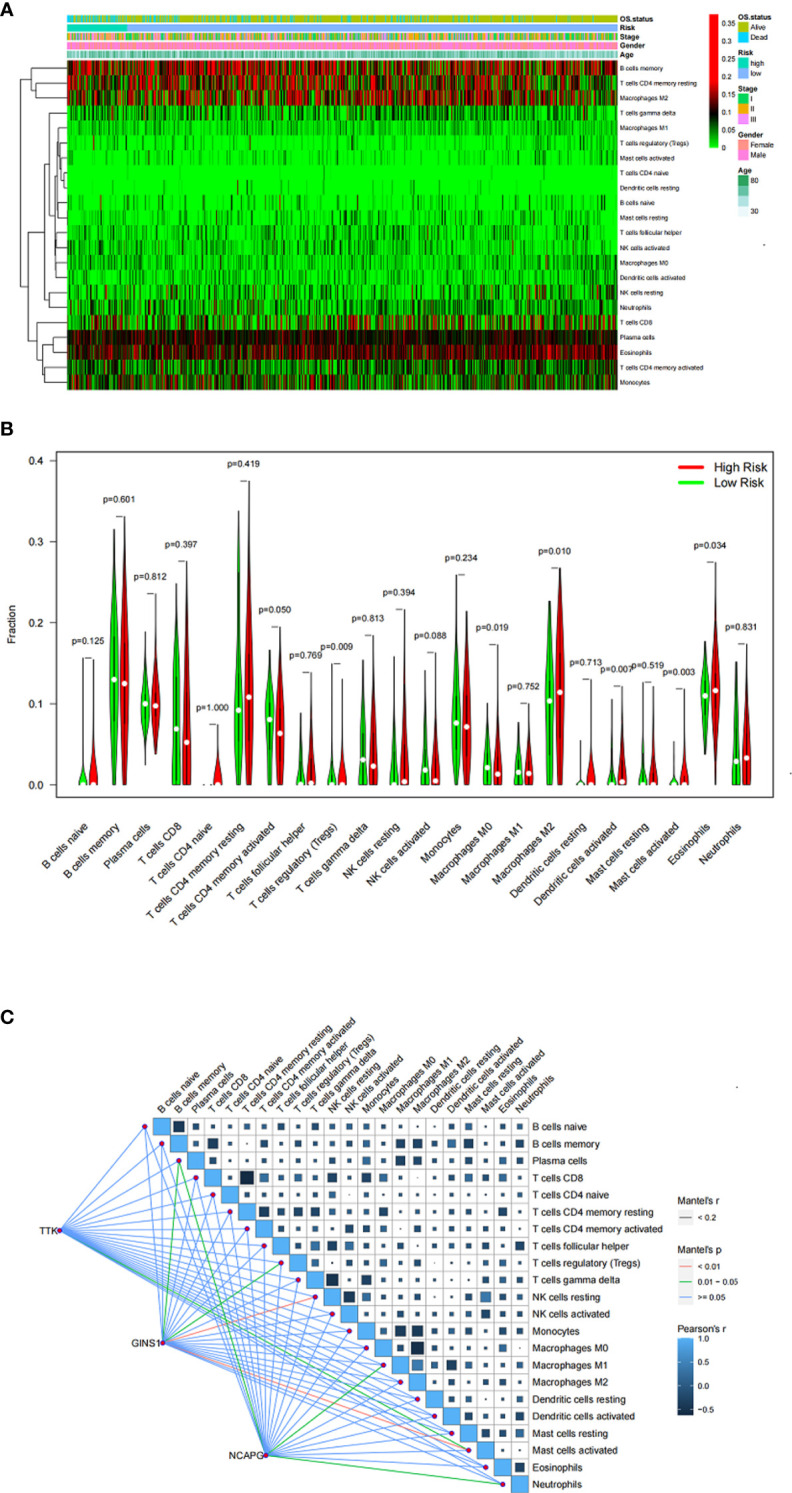
**(A)** Heatmap showing the proportion of TIICs in MM samples. **(B)** Violin plot showing the ratio differentiation of TIICs between MM samples with low or high risk. **(C)** Correlation of the expression of three genes with the expression of TIICs.

The differential abundance of TIICs is shown in the heatmap (**Figure 6A**). Six TIICs (activated memory CD4 T cells, M0 macrophages, M1 macrophages, eosinophils, activated dendritic cells, and activated mast cells) showed significant differences between different risk groups (**Figure 6B**). The results also showed that the 3 genes (TTK, GINS and NCAPG) were correlated with TIICs (**Figure 6C**).

### GSEA

**Figure 7:**
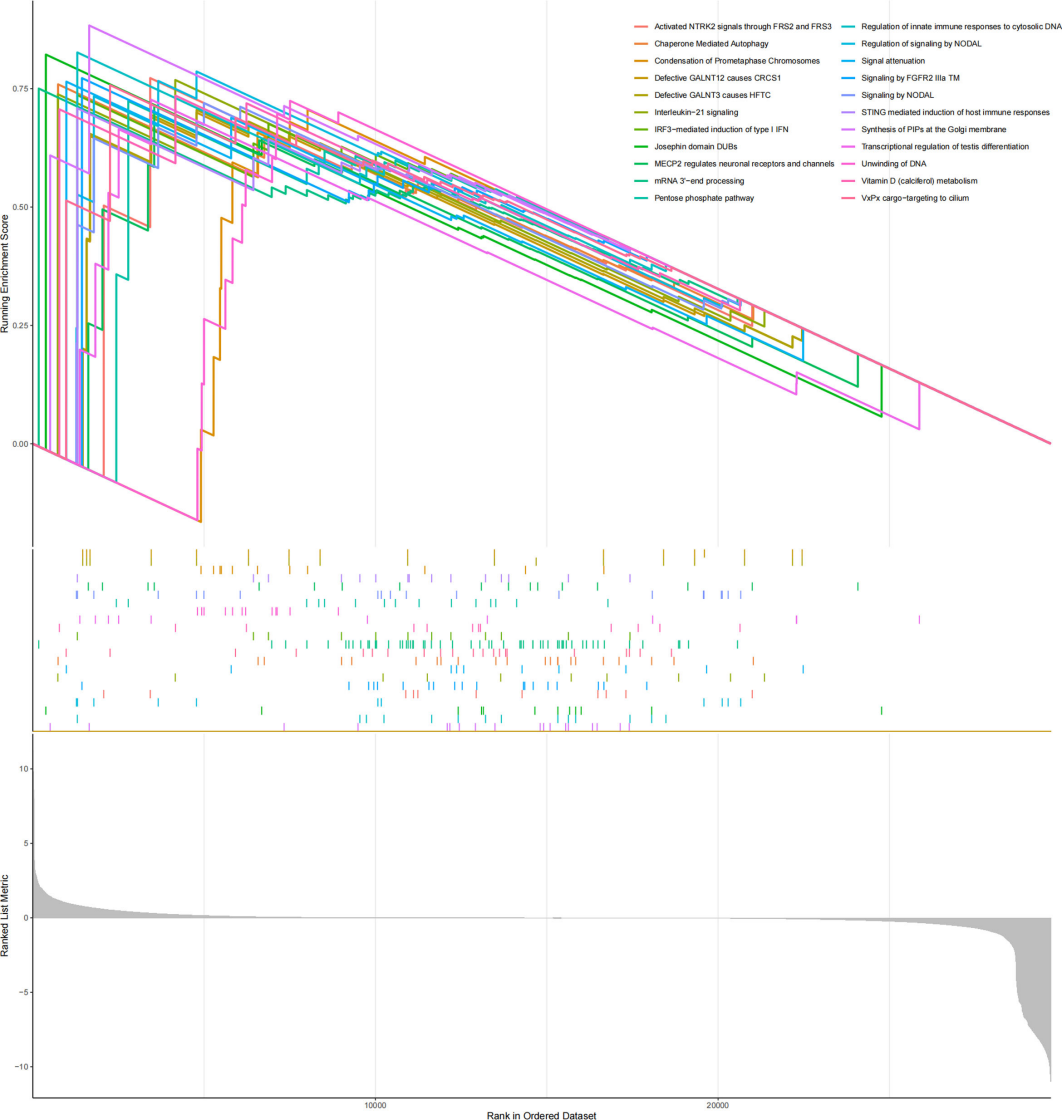
GSEA results showed that the reactome pathway was enriched in MM samples from the high-risk group.

Reactome GSEA pathway interaction analysis revealed that 22 significant pathways were dramatically changed in the high-risk group (**Figure 7**), involving cancer, the immune system, metabolism, and signal transduction.

### Suitable drugs for high-risk patients

**Figure 8:**
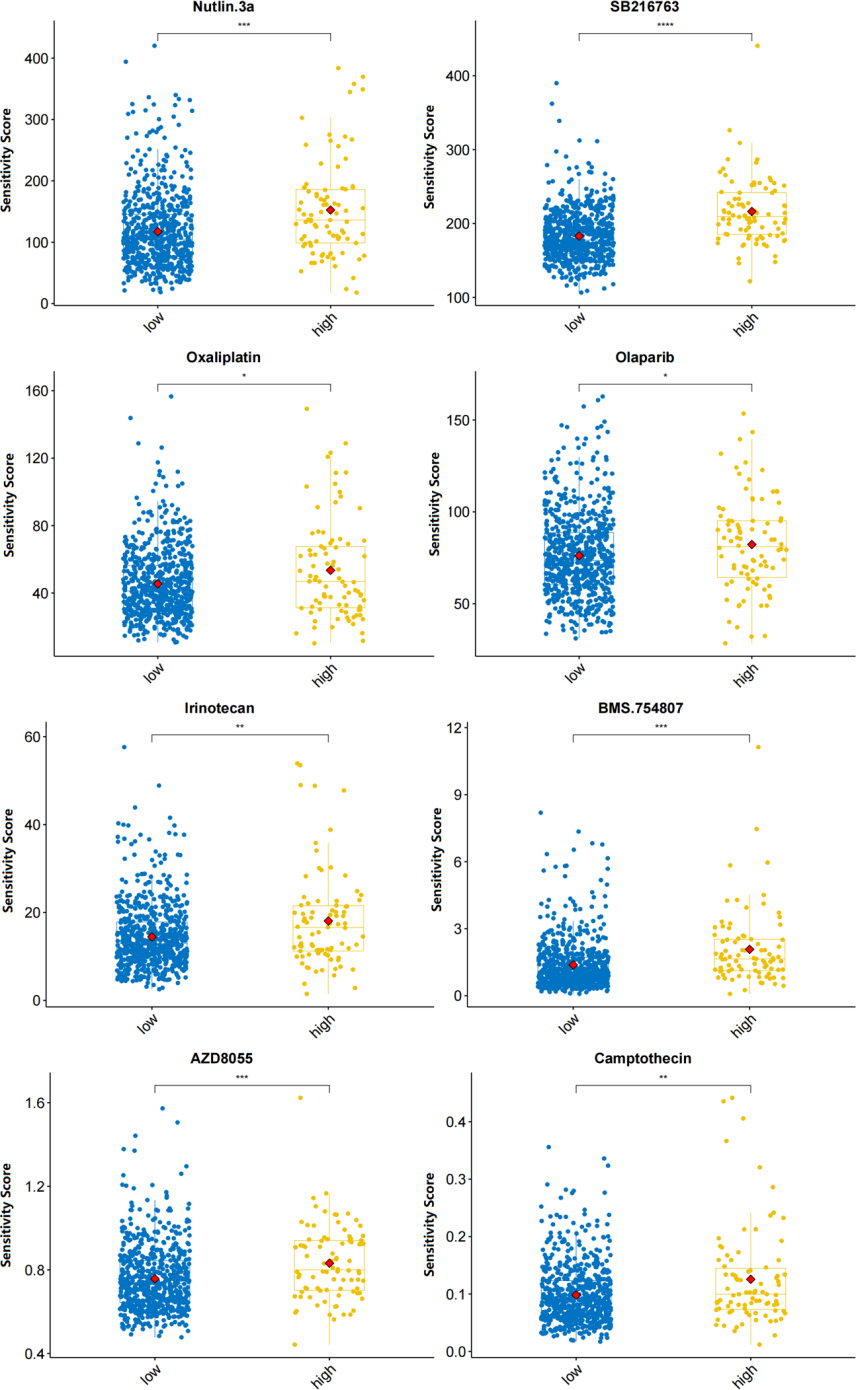
Predicted sensitivity scores of drugs that are candidate therapeutic agents for high-risk MM patients (*p<0.05, **p<0.01, ***p<0.001, ****p<0.0001).

The OncoPredict algorithm showed that high-risk MM patients were more sensitive to 8 drugs, including Nutlin-3a (MDM2 inhibitor), SB216763 (GSK3 inhibitor), oxaliplatin (platinum anticancer drug), olaparib (PARP inhibitor), irinotecan (TopoI inhibitor), BMS-754807 (IGF-1R/IR inhibitor), AZD8055 (mTOR inhibitor), and camptothecin (TopoI inhibitor) (**Figure 8**).

## Discussion

An in-depth understanding of the biological mechanisms underlying carcinogenesis in MM is vital to find strategies for MM treatment. To the best of our knowledge, there are few studies on the role of three genes (TTK, GINS1, and NCAPG) in MM. TTK is an integral part of the spindle assembly checkpoint, which is responsible for maintaining the integrity of the genome (16). TTK is hardly detectable in normal tissues except the testis and placenta. TTK is an indicator of poor prognosis in breast cancer (17), hepatocellular carcinoma (18), lung cancer (19), and glioma (20). Inhibition of TTK could induce MM cell (AMO-1) apoptosis and deregulate the proliferation *in vitro* (21). GINS1 is a member of the GINS complex that plays a vital role in DNA replication (22). GINS1 was found to be related to poor prognosis in breast and liver cancer (23, 24). GINS1 physically interacts with TOP2A (25), which might be a highly significant predictor of response to proteasome inhibitors (26). NCAPG, a subunit of condensin I, is responsible for the condensation or structure of mitotic chromosomes (27). The expression level of NCAPG has been shown to be closely related to the prognosis of tumors (28–31). NCAPG involves in the regulation of different signaling pathways, such as PI3K/AKT (32), NF-κB (33) and SRC/STAT3 (34) signaling pathway. However, the effects of GINS1 and NCAPG in MM remain unknown.

The CIBERSORT algorithm was used to estimate immune cell infiltration in the bone marrow (BM) microenvironment. The proliferation, progression, and survival of malignant plasma cells (PCs) in MM are highly affected by the BM microenvironment (35). BM microenvironment is highly enriched for suppressive immune cells such as MDSCs, Tregs, pDCs, Bregs, N2 neutrophils, M2 macrophages, which leads to effector cell dysfunction and lack of persistence (36). We found that M2 macrophages and regulatory T cells (Tregs) had apparent abnormal infiltration in MM, contributing to the immune evasion, proliferation, and drug resistance of MM cells (37). We also found that these immunosuppressive cells are in bidirectional and multidirectional crosstalk inhibited memory effector T-cell populations. Eosinophils (Eos) can contribute to the proliferation of malignant PCs in MM (38). Suzuki et al. study showed that the median time to next treatment (TTNT) in the elevated eosinophil group was significantly longer than that in the nonelevated group (40.3 months *vs* 8.4 months; P = .017) in relapsed or refractory myeloma patients treated with lenalidomide (39). Mast cell (MC) accumulation correlates with increased neovascularization in MM (40). Raised IL-6 levels can be caused by the presence of MC (41), and it has been identified as the key growth and survival factor for myeloma cells (42). Tumor lesions in MM are highly infiltrated by dendritic cells (43). TTK could affect the tumor microenvironment (TME) by affecting the number of immune cells (44). No literature is available on the role of GINS1 and NCAPG in the TME.

Reactome GSEA pathway interaction analysis revealed that several pathways were dramatically changed in the high-risk myeloma patients. The IL-21 signaling pathway in myeloma cells involves phosphorylation of Erk1/2, Jak1, and Stat3 (45). The upregulation of chaperone-mediated autophagy (CMA) is a potential mechanism of resistance to bortezomib (46). The pentose phosphate pathway protects against oxidative stress-mediated late apoptosis/necrosis of multiple myeloma cells (47).

Our study showed that 8 drugs were effective in high-risk MM patients. Nutlin-3 can disrupt the p53-MDM2 interaction and activate p53. Nutlin-3 with bortezomib may increase clinical responsiveness to bortezomib-based therapy (48). The chemical inhibitor SB216763 leads to a reduction in MM cell growth and augments the response of MM cells to the cytotoxic effects of bortezomib (49). Oxaliplatin triggers bona fide “immunogenic cell death” (ICD), as it provokes a premortem endoplasmic reticulum stress response (50), so it can be used as an inducer of ICD and as a modulator of the TME (51). Drugs that induce excessive amounts of ER stress, such as proteasome inhibitors and novel ER stressors, are predicted to be very effective in targeting MM cells (52). The PARP1 inhibitor olaparib impaired MM cell viability *in vitro* and was effective against MM *in vivo* xenografts (53). Efficacy needs to be further evaluated in clinical trials.

Certainly, our research still has some limitations. First, only transcriptome data were included for the MM prognosis study. Second, some clinical data are missing, such as cytogenetic profile and type of measurable disease. Third, the carcinogenic mechanism of the three genes in MM warrants intensive study. Fourth, we used algorithm analyses to predict drug sensitivity but did not verify it.

In conclusion, our findings may improve the understanding of the factors that influence development and prognosis in MM, which may present a new strategy.

## Data availability statement

The datasets presented in this study can be found in online repositories. The names of the repository/repositories and accession number(s) can be found in the article/supplementary material.

## Ethics statement

Ethical review and approval was not required for the study on human participants in accordance with the local legislation and institutional requirements. Written informed consent for participation was not required for this study in accordance with the national legislation and the institutional requirements.

## Author contributions

JW and YW conceived and designed the study. JW, LG, CL, and MZ performed the electronic search, data collection, abstraction, and data entry. JW, LG, MZ, and CL were statistical advisers. JW, and YW were responsible for the overall direction of the text and discussion. JW and YW had full access to all the data in the study and takes responsibility for the integrity of the data and the accuracy of the data analysis. All authors contributed to the article and approved the submitted version.
